# Eschar, enigma, and encephalopathy: a case series of atypical and fatal presentations of scrub typhus in young adults from Northern India

**DOI:** 10.17179/excli2026-9514

**Published:** 2026-06-27

**Authors:** Abhijeet Sharma, Yashendra Sethi, Shirobhi Sharma, Akansha Jain, Prakhar Choudhry, Nidhi Uniyal, Pankaj N. Choudhry, Shivani Chopra, Hitesh Chopra, Pratik Agarwal

**Affiliations:** 1 Department of Research, PearResearch, Dehradun, India; 2 Department of Medicine, Subharti Medical College, Swami Vivekanand Subharti University, Meerut, India; 3 Vigyaved Healthcare, Dehradun, India; 4 Moti Lal Nehru Medical College, Prayagraj, India; 5 Graphic Era Institute of Medical Sciences, Dehradun, India; 6 Max Superspeciality Hospital Vaishali, Ghaziabad, India; 7 Department of Biosciences, Saveetha School of Engineering, Saveetha Institute of Medical and Technical Sciences, Chennai - 602105, Tamil Nadu, India; 8 Centre for Research Impact & Outcome, Chitkara College of Pharmacy, Chitkara University, Rajpura, 140401, Punjab, India; 9 Department of Psychiatry, Shikha ‘O’ Anusandhan University, Bhubaneshwar, India

**Keywords:** Scrub typhus, acute kidney injury, acute respiratory distress syndrome, acute encephalitis syndrome, eschar

## Abstract

Scrub typhus is a re-emerging but often underdiagnosed tropical zoonosis in India. Its clinical heterogeneity, serological overlap with other endemic infections, and frequent absence of pathognomonic signs contribute to delayed diagnosis and high complication rates. Central nervous system, pulmonary, and renal involvement signify severe disease and are associated with considerable morbidity and mortality. We describe three cases of previously healthy young adult males (ages 24-32) who presented with severe, organ-threatening complications of scrub typhus: acute encephalitis syndrome (AES), acute respiratory distress syndrome (ARDS), and acute kidney injury (AKI). All patients had evidence of systemic inflammation, thrombocytopenia, and multiorgan dysfunction. Eschars-although present in all cases-were initially missed or overlooked, delaying causal diagnosis. Serologic cross-reactivity with dengue, chikungunya, and leptospira further complicated early recognition. Despite early initiation of doxycycline upon clinical suspicion, one patient with ARDS succumbed to progressive hypoxemia and multiorgan failure. These cases underscore the protean and fulminant nature of scrub typhus and highlight the diagnostic importance of thorough skin examination in febrile patients with unexplained CNS, pulmonary, or renal involvement. In endemic settings, empiric doxycycline should be considered early, even in the absence of classical signs. Early recognition and treatment remain the most critical determinants of survival. Our experience reinforces that scrub typhus, though elusive, is a potentially treatable cause of severe febrile illness when promptly identified.

## Introduction

Scrub typhus is an acute febrile illness caused by *Orientia tsutsugamushi*, an obligate intracellular Gram-negative bacterium transmitted to humans through the bite of infected chiggers-the larval stage of trombiculid mites. Endemic to the "tsutsugamushi triangle," which spans South and Southeast Asia, northern Australia, and the western Pacific, scrub typhus has seen a marked resurgence in India over the past two decades. While classically considered a disease of forested or rural areas, increasing reports from urban and peri-urban regions reflect changing epidemiology, likely driven by climate variability, unregulated land use, and improved clinician awareness (Prakash, 2017[11]). In India, the burden is especially pronounced in the sub-Himalayan belt, northeast states, and northern plains, including Uttar Pradesh, where outbreaks have become increasingly common during the post-monsoon months. India has witnessed a resurgence of cases in both rural and peri-urban settings, driven by climate variability, land-use changes, and increased awareness (Prakash, 2017[11]; Singh and Panda, 2024[14]; Tsay and Chang, 1998[16]).

Clinically, scrub typhus is notoriously deceptive. Although the pathognomonic triad of fever, rash, and eschar is well described, it is infrequently seen in full-especially in Indian patients. Instead, scrub typhus typically presents as a nonspecific febrile illness resembling other endemic infections such as dengue, leptospirosis, malaria, or enteric fever, often leading to delayed or inappropriate treatment (Singh and Panda, 2024[14]). The absence of distinguishing features on initial presentation means that patients are frequently misdiagnosed or empirically treated for alternate infections unless a high index of suspicion is maintained.

The pathophysiology of scrub typhus is driven by bacterial replication within endothelial cells, leading to widespread small-vessel vasculitis, perivasculitis, and capillary leak. This mechanism underlies the diverse and potentially fatal complications associated with the disease, including acute respiratory distress syndrome (ARDS), acute kidney injury (AKI), myocarditis, hepatitis, meningoencephalitis, and multiorgan dysfunction syndrome (MODS). Importantly, these complications may emerge abruptly and in isolation, without classical prodromal signs, thus masquerading as isolated organ dysfunction of alternate etiologies (Tsay and Chang, 1998[16]). The central nervous system, pulmonary parenchyma, and renal microvasculature are particularly vulnerable to this inflammatory injury, and their involvement significantly increases morbidity and mortality.

A potentially lifesaving clue is the eschar-a painless, blackened necrotic lesion at the site of mite inoculation. However, eschars are often missed due to their small size, painless nature, and predilection for covered or inconspicuous sites such as the thigh, groin, axilla, inframammary folds, or perineum. In dark-skinned individuals, their detection may be even more challenging. Eschar prevalence varies by geography and patient population but is reported in only 10-50 % of Indian cases, further complicating timely diagnosis (Jamil et al., 2014[7]; Singh and Panda, 2024[14]; Tsay and Chang, 1998[16]). Nevertheless, when present, an eschar provides a critical diagnostic clue that should prompt early empirical therapy with doxycycline-especially in endemic settings. The reference diagnostic test is the indirect immunofluorescence assay (IFA), though limited availability in India necessitates reliance on IgM ELISA, Weil-Felix, or rapid immunochromatographic tests (Jamil et al., 2014[7]; Saxena et al., 2014[13]). Polymerase chain reaction (PCR) offers high specificity but is seldom accessible in routine clinical care. Each method has limitations, including cross-reactivity with dengue, leptospira, and chikungunya, complicating early recognition. Further, most published series describe elderly, immunocompromised, or rural populations. Our report adds a unique perspective by documenting fulminant disease in previously healthy young adults, each with a different life-threatening complication, in whom eschars were initially overlooked and serologic overlap led to misdiagnosis.

We describe three illustrative cases of complicated scrub typhus in young men admitted to a tertiary care center in Western Uttar Pradesh. Each patient presented with a distinct but severe complication-acute encephalitis syndrome (AES), ARDS, and AKI-underscoring the protean nature of this infection. All three had delayed or missed eschars and overlapping features with other endemic febrile illnesses. These cases not only exemplify the clinical severity of untreated scrub typhus but also emphasize the need for high clinical suspicion, meticulous physical examination, and early empirical therapy to reduce the risk of irreversible organ injury or death.

***Key learning points***

-------

**What is already known on this topic:**

- Scrub typhus is a re-emerging zoonotic disease in South and Southeast Asia, often presenting as a nonspecific febrile illness.
- Its severe manifestations-including ARDS, AKI, and meningoencephalitis-are linked to small-vessel vasculitis caused by *Orientia tsutsugamushi*.
- Eschar, while pathognomonic, is inconsistently present and often missed in clinical practice.
- Early initiation of doxycycline dramatically improves prognosis, yet diagnostic delays remain common due to overlapping clinical presentations with other endemic infections such as dengue, leptospirosis, and malaria.

-------

**What this study adds:**

- This case series highlights three fulminant presentations of scrub typhus in previously healthy young adults, each with a different form of organ failure-neurologic (AES), pulmonary (ARDS), and renal (AKI).
- All three patients had eschars that were either overlooked or discovered late, reinforcing the need for thorough physical examination in febrile patients in endemic regions.
- The series underscores that even in the absence of classical signs or confirmatory tests, empiric doxycycline should be initiated early in patients with undifferentiated febrile illness and systemic inflammation
- Clinical outcomes strongly correlate with the timing of antibiotic initiation and supportive care, even in patients requiring intensive care.

-------

**Key lessons for clinical practice:**

- **Maintain high suspicion** for scrub typhus in any febrile illness with unexplained CNS, respiratory, or renal involvement in endemic areas, especially post-monsoon.
- **Perform a meticulous search for eschar**, particularly in concealed areas, regardless of patient age or disease severity.
- **Initiate empirical doxycycline promptly**, even before laboratory confirmation, as early treatment is the single most critical determinant of outcome.
- Recognize the potential for severe disease in young, immunocompetent individuals, and do not underestimate scrub typhus based on patient demographics.
- **Integrate scrub typhus into syndromic surveillance and fever algorithms**, especially in areas with rising incidence and poor access to confirmatory diagnostics.

-------

## Case Presentation

### Case 1: Neuroinvasive scrub typhus presenting as acute encephalitis syndrome in a young male misdiagnosed as dengue encephalitis

A 24-year-old male, previously healthy and employed as a college student in Meerut, Uttar Pradesh, presented to the emergency department of Chhatrapati Shivaji Subharti Hospital with complaints of high-grade fever for three days, altered mental status since the same duration, and the patient experienced two generalized tonic-clonic seizures within 24 hours, followed by postictal drowsiness.. The febrile episode was abrupt in onset, rapidly progressive, and continuous in nature, with no observed diurnal variation, chills, or rigor. The fever responded transiently to over-the-counter antipyretics but returned within hours. Notably, the patient's caregivers reported that he had become increasingly confused and irritable by the second day of illness, and on the third day, he had a witnessed episode of generalized tonic-clonic seizure with associated tongue biting, ocular up-rolling, and a postictal phase characterized by profound drowsiness. A second similar episode occurred within six hours, followed by a period of unresponsiveness lasting approximately two hours.

Before presentation to our center, the patient had been evaluated at a peripheral clinic where an NS1 antigen test for dengue virus returned positive. On this basis, a diagnosis of dengue encephalitis was presumed, and supportive care, including intravenous fluids and oral levetiracetam, was initiated. However, persistent neurological deterioration prompted referral to our tertiary care facility.

**Figure 1:**
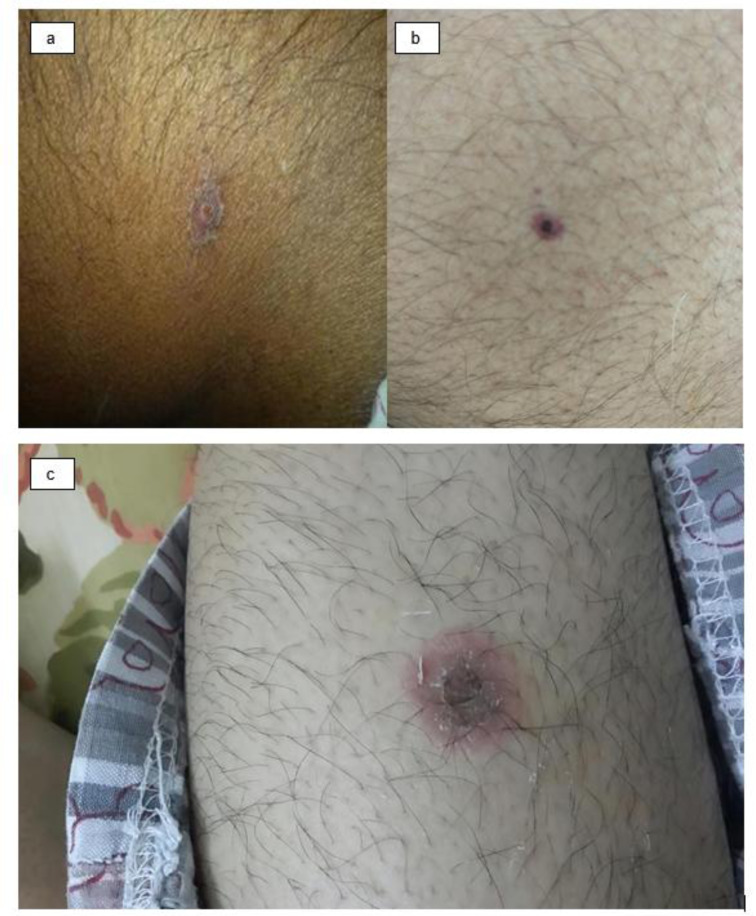
Figure 1a, b, c: Eschar of Scrub Typhus for patients 1,2 & 3

At presentation, the patient was febrile (101.6 °F), with a blood pressure of 110/70 mm Hg, heart rate of 98 beats/min, and respiratory rate of 20 breaths/min. Oxygen saturation on room air was 97 %. Neurologically, the patient had a Glasgow Coma Scale (GCS) score of E3V4M5. Pupils were equal and reactive. There was no neck stiffness, photophobia, Kernig's or Brudzinski's sign. Plantar responses were flexor bilaterally. Fundus examination did not reveal papilledema. A thorough systemic examination was otherwise unremarkable. However, careful inspection revealed a solitary necrotic lesion, approximately 1.5 cm in diameter with surrounding erythema, in the right axillary fold-morphologically consistent with an eschar (Figure 1a, b and c(Fig. 1)).

Initial laboratory investigations revealed thrombocytopenia (platelet count 78,000/μL), mild leukocytosis (WBC count 11,800/μL), and an elevated high-sensitivity C-reactive protein (hsCRP) level of 44 mg/L. Renal and liver function tests were within normal limits. Electrolytes and blood glucose levels were unremarkable. Coagulation profile showed a mildly prolonged activated partial thromboplastin time (aPTT), but prothrombin time and INR were normal.

Given the presence of seizures, altered sensorium, and fever, a diagnosis of acute encephalitis syndrome (AES) was provisionally considered. A diagnostic lumbar puncture was performed after ensuring absence of papilledema. Cerebrospinal fluid (CSF) analysis showed normal opening pressure, normal glucose (66 mg/dL), protein (36 mg/dL), and no pleocytosis. CSF Gram stain, acid-fast bacilli stain, and India ink preparation were negative. PCR for herpes simplex virus (HSV-1 and 2), Japanese encephalitis virus, and enteroviruses returned negative results.

**Figure 2:**
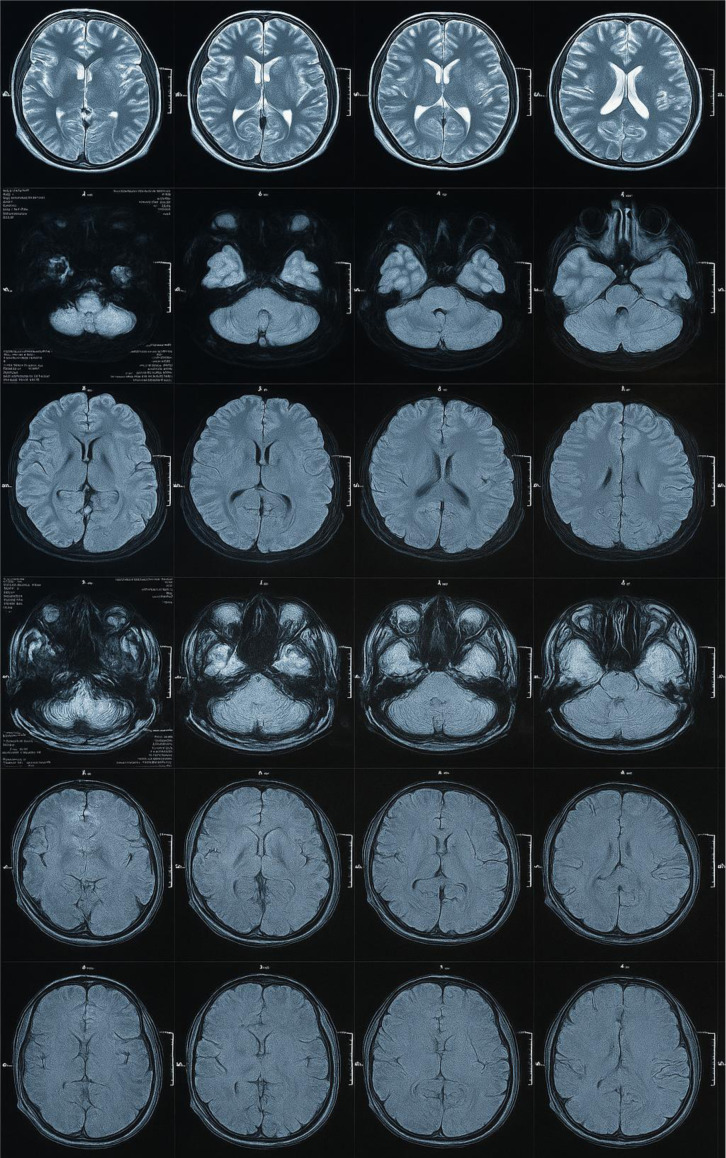
MRI Brain (FLAIR and T2 Axial Sequences) in a Case of Scrub Typhus Encephalitis. MRI brain axial sequences (FLAIR and T2-weighted images) showing suggestive findings of scrub typhus encephalitis - the scans reveal bilateral, symmetric hyperintense lesions predominantly involving the thalami, basal ganglia, and brainstem-especially the midbrain and pons. Subtle involvement of the cerebellar peduncles and periventricular white matter is also noted. No significant mass effect, midline shift, or hemorrhagic transformation is present.

Magnetic resonance imaging (MRI) of the brain was performed on day 2 of admission and revealed bilateral symmetrical hyperintensities in the thalamic regions on FLAIR and T2-weighted sequences without any associated mass effect or diffusion restriction. These findings, though not pathognomonic, have been previously reported in patients with rickettsial CNS involvement and are considered highly suggestive of scrub typhus encephalitis in endemic areas (Figure 2(Fig. 2)) (Prakash, 2017[11]; Singh and Panda, 2024[14]).

Given the axillary eschar and endemicity of scrub typhus in the region, serum IgM ELISA for *Orientia tsutsugamushi* was ordered and returned strongly positive. Interestingly, repeat serology for dengue showed positive IgG but negative IgM-suggesting past exposure rather than an active infection. NS1 positivity in this context was likely a false positive, as cross-reactivity with rickettsial antigens has been previously reported (Tsay and Chang, 1998[16]). Blood and urine cultures were sterile.

A definitive diagnosis of scrub typhus-associated AES was made. Intravenous doxycycline (100 mg twice daily) was initiated immediately. Seizure control was maintained with levetiracetam, and supportive care included intravenous fluids, paracetamol for fever, and head elevation. Empiric antiviral therapy (acyclovir) was discontinued after negative HSV PCR.

The patient began to show clinical improvement by day 4 of doxycycline. Fever resolved, sensorium gradually improved, and repeat neurological assessment on day 6 showed a GCS of E4V5M6. Platelet count improved to 112,000/μL. A repeat MRI on day 8 showed partial resolution of thalamic lesions. He was discharged on day 9 of hospitalization with complete neurological recovery and advised to complete a 10-day oral doxycycline course.

This case underscores several important diagnostic and therapeutic lessons. First, in endemic regions of India, scrub typhus should remain high on the differential for AES, even when preliminary serology suggests arboviral infections like dengue or chikungunya. Second, the presence of a pathognomonic eschar, though found in less than 50 % of Indian patients (Jamil et al., 2014[7]), is an invaluable diagnostic clue and should prompt immediate reconsideration of rickettsial disease. Third, suggestive MRI findings-especially bilateral thalamic hyperintensities-should alert clinicians to neuroinvasive scrub typhus, as has been previously described in Southeast Asian literature (Singh and Panda, 2024[14]; Saxena et al., 2014[13]).

Despite the normal CSF findings, CNS involvement in scrub typhus is not uncommon. Inflammatory damage occurs due to small-vessel vasculitis affecting the cerebral microvasculature rather than direct pathogen invasion, explaining the paucity of CSF abnormalities in many cases (Mohanty et al., 2019[10]). Prompt initiation of doxycycline is critical; delays of more than 5-7 days after symptom onset are associated with increased risk of neurologic sequelae and death (Devasagayam et al., 2021[4]).

This case also highlights the limitations of relying solely on NS1 and IgG/IgM panels in the diagnostic algorithm for febrile encephalopathy. Serologic cross-reactivity, variable timing of antigenic expression, and test heterogeneity must all be considered in resource-limited settings where empirical therapy may be warranted.

### Case 2: Fatal illness consistent with scrub typhus-associated ARDS with septic shock

A 24-year-old male presented with fever and progressive shortness of breath for 5 days. He had no past medical history. His family reported that three days prior to symptom onset, he had spent a day clearing vegetation at a peri-urban plot that the family was considering purchasing. Two days later, he developed high-grade fever, which remained unrelenting despite over-the-counter medications. On day three of illness, he complained of breathlessness that progressed from exertional to at-rest dyspnea over 48 hours. He presented to the emergency department in respiratory distress.

At admission, the patient was febrile (102.2 °F), tachypneic (RR 38/min), with oxygen saturation of 78 % on room air. Pulse was 116/min, BP was 96/60 mmHg. On auscultation, bilateral coarse crepitations were present. No rash or lymphadenopathy was evident. A 2 cm blackish eschar was found in the thigh, surrounded by erythema, but it was noted only after a second thorough skin examination.

Laboratory tests revealed leukocytosis (TLC 33,000/μL), severe thrombocytopenia (platelets 9,000/μL), mild transaminitis, and normal renal function. Arterial blood gas showed PaO₂/FiO₂ ratio <100, consistent with severe ARDS. Chest X-ray demonstrated bilateral homogeneous alveolar opacities (Figure 2(Fig. 2)). Scrub typhus IgM was positive by ELISA. Leptospira IgM and chikungunya IgG were also positive. While these results were considered clinically inconsistent and likely representing cross-reactivity (Tsay and Chang, 1998[16]), the possibility of co-infection or an alternative primary diagnosis like leptospirosis could not be definitively ruled out due to the serological overlap.

He was started on IV doxycycline (100 mg twice daily), IV ceftriaxone, and oseltamivir. He was intubated and placed on pressure-controlled ventilation. Despite appropriate sedation and PEEP titration, oxygenation remained poor. Prone positioning was initiated on day 2 of ICU stay, after which transient improvement in saturation (from 84 % to 91 %) was observed. Two units of single donor platelets were administered. Blood cultures remained sterile.

However, on day 4, the patient developed hypotension requiring vasopressors. Sequential Organ Failure Assessment (SOFA) score was 12. On day 5, he experienced a desaturation episode despite high FiO₂, followed by cardiac arrest. Resuscitative efforts failed.

Death was consistent with fulminant illness attributed to scrub typhus-associated ARDS and septic shock. However, given the overlapping positive serologies for co-endemic pathogens (Leptospira IgM and Chikungunya IgG) and the absence of an autopsy, a definitive causal attribution is limited, and a severe co-infection or an alternative primary diagnosis cannot be fully excluded.

This case underlines the fulminant potential of pulmonary involvement in scrub typhus. ARDS is among the most lethal complications, with mortality rates approaching 25-30 % even with ICU-level care (Jamil et al., 2014[7]; Saxena et al., 2014[13]). Coinfections may delay correct treatment if clinical correlation is lacking. The eschar was only found on re-examination-underscoring the need for meticulous skin inspection.

### Case 3: Acute kidney injury secondary to scrub typhus-associated vasculitis and hypoperfusion

A 32-year-old male manual laborer from rural western Uttar Pradesh presented with a five-day history of high-grade fever, generalized malaise, and progressively decreasing urine output. The fever was intermittent, associated with chills and myalgia, but without rash, vomiting, diarrhea, cough, dyspnea, or altered sensorium. He denied any hematuria, flank pain, dysuria, or urinary urgency. There was no history of recent travel, medication use (including NSAIDs or antibiotics), insect or snake bites, exposure to industrial toxins, or known contact with jaundiced individuals. He had not been vaccinated recently and had no prior history of diabetes, hypertension, renal disease, or autoimmune illness.

The patient had initially sought care at a local clinic, where he received paracetamol and oral rehydration, but his symptoms worsened over the next two days, with progressive fatigue and darkening of urine. On presentation to our hospital, he appeared dehydrated and mildly obtunded. Vital signs revealed a temperature of 101.4 °F (38.6 °C), hypotension (blood pressure 92/60 mmHg), tachycardia (heart rate 108 bpm), and a respiratory rate of 20/min with oxygen saturation of 98 % on room air. He had dry mucous membranes, reduced skin turgor, and delayed capillary refill time. Importantly, there were no peripheral stigmata of infective endocarditis, no rash or petechiae, no palpable lymphadenopathy, no hepatosplenomegaly, and no peripheral edema. Cardiopulmonary and neurologic examinations were within normal limits.

A meticulous skin examination revealed a 1.5 cm ulcerative eschar with a dry necrotic center and surrounding erythema in the left thigh region-a pathognomonic clue often missed in dark-skinned individuals or obscured in concealed areas. The remainder of systemic examination was unremarkable.

Laboratory investigations showed: serum creatinine 4.0 mg/dL, urea 130 mg/dL, sodium 132 mEq/L, potassium 3.8 mEq/L, leukocyte count 12,400/μL, platelet count 112,000/μL, and normal liver transaminases. Urinalysis revealed trace proteinuria without hematuria, pyuria, or cellular casts. Blood cultures were sterile, and rapid diagnostic tests for malaria, dengue, and leptospirosis were negative. Scrub typhus IgM by ELISA was positive. Renal ultrasonography showed normal-sized kidneys with preserved corticomedullary differentiation and no signs of obstruction or cortical scarring.

A diagnosis of scrub typhus-associated acute kidney injury (AKI), likely due to prerenal azotemia with evolving acute tubular necrosis (ATN), was made. The patient was promptly initiated on intravenous doxycycline (100 mg twice daily), along with aggressive volume repletion using isotonic saline and Ringer's lactate, targeting urine output and hemodynamic stability. Empirical broad-spectrum antibiotics were withheld pending culture results.

Over the next 24 hours, the patient remained oliguric but normotensive. Gradually, he demonstrated a positive fluid balance with steady diuresis beginning on day 2. Serum creatinine fell to 2.0 mg/dL by day 4 and normalized (1.3 mg/dL) by day 7. Platelet counts remained stable, and he became afebrile by day 3 of doxycycline therapy. Improvement was likely due to the combined effect of doxycycline and supportive therapy, including fluids and renal care. No dialysis was required. He was discharged on day 9 in stable condition with complete recovery of renal function. Table 1-5 display the summary of the three cases including their baseline demographics (Table 1(Tab. 1)), clinical features and organ involvement (Table 2(Tab. 2)), laboratory parameters (Table 3(Tab. 3)), imaging and CSF findings (Table 4(Tab. 4)) as well as treatment and clinical course (Table 5(Tab. 5)).

## Discussion

The novelty of this study lies in documenting three distinct, fulminant, and organ-threatening presentations of scrub typhus-AES, ARDS, and AKI-specifically within previously healthy young adults. This cohort provides a contrast to existing literature, which primarily focuses on older, rural, or immunocompromised populations.

These three cases represent the protean and potentially fatal spectrum of complicated scrub typhus in young Indian males, reinforcing the pathogen's capacity to precipitate multisystem failure through widespread small-vessel vasculitis and endothelial dysfunction (Bonell et al., 2017[2]; Devasagayam et al., 2021[4]; Mohanty et al., 2019[10]; Saxena et al., 2014[13]; Varghese et al., 2023[17]). Beyond endothelial damage, immune dysregulation and cytokine storm underpin life-threatening complications such as acute encephalitis syndrome (AES), acute respiratory distress syndrome (ARDS), and acute kidney injury (AKI) (Behera et al., 2019[1]; Kispotta et al., 2020[9]; Taylor et al., 2015[15]).

Timely diagnosis remains a formidable challenge due to the clinical and serological overlap with other endemic tropical illnesses like dengue, leptospirosis, malaria, and chikungunya (Singh and Panda, 2024[14]; Jamil et al., 2014[7]). This diagnostic ambiguity is exacerbated by the lack of reference-standard assays in peripheral settings, often necessitating reliance on less sensitive tools prone to cross-reactivity. In this context, the pathognomonic eschar remains an invaluable clinical clue. Although present in only 1050 % of Indian patients, its detection should prompt immediate presumptive therapy, especially when accompanied by systemic markers like thrombocytopenia or multiorgan involvement (Jamil et al., 2014[7]; Saxena et al., 2014[13]; Mohanty et al., 2019[10]).

Early initiation of doxycycline preferably within the first 5 days of illness is the single most critical determinant of survival (Prakash, 2017[11]; Saxena et al., 2014[13]). Delayed therapy significantly increases the risk of irreversible organ damage and death, particularly when neurological or pulmonary complications emerge (Devasagayam et al., 2021[4]; Kannan et al., 2020[8]). Our cohort reinforces this urgency: while neurologic and renal cases recovered with prompt treatment, the fatal outcome in the ARDS case underscores the peril of delayed recognition in a setting of overlapping symptoms and late clinical presentation.

Doxycycline remains the cornerstone of therapy for scrub typhus. Its efficacy in reducing mortality is highest when administered early-preferably within the first 5 days of illness (Prakash, 2017[11]; Saxena et al., 2014[13]). Delayed therapy, especially beyond the onset of respiratory or neurologic complications, significantly increases the risk of death. In our series, empiric doxycycline was initiated upon clinical suspicion, guided by endemicity and eschar presence, aligning with WHO and Indian Ministry of Health guidelines; early treatment is the key determinant of survival. Recent evidence from a pivotal multicenter trial has demonstrated that combination therapy with intravenous doxycycline and azithromycin may provide superior outcomes in severe cases (El Sayed et al., 2018[5]; Griffith et al., 2014[6]; Rajapakse et al., 2017[12]; Saxena et al., 2014[13]). Varghese et al. (2023[17]) showed that dual therapy reduced treatment failure rates and accelerated clinical resolution in hospitalized patients with complications such as ARDS or myocarditis (Varghese et al., 2023[17]). This strategy, though not yet universally adopted, merits consideration in ICU settings where mortality risk is high (Mohanty et al., 2019[10]; Saxena et al., 2014[13]; Varghese et al., 2014[18]).

The role of macrolides, particularly azithromycin, has gained prominence not only due to its favorable safety profile in pregnancy and children, but also for its efficacy in doxycycline-refractory or contraindicated scenarios (Devasagayam et al., 2021[4]). A Cochrane review by El Sayed et al. (2018[5]) affirmed that both doxycycline and azithromycin are effective, but called for higher-quality evidence to guide therapy in critically ill subgroups (El Sayed et al., 2018[5]). Cai and Fang (2024[3]) have further underlined the importance of prompt identification and aggressive treatment, citing case reports where delayed diagnosis led to irreversible end-organ damage and death-even in patients initially appearing stable (Cai and Fang, 2024[3]). The pathophysiology and management of these atypical, organ-specific manifestations are categorized in Table 6(Tab. 6).

Critical care interventions remain adjunctive but essential. Our experience reiterates the value of prone ventilation and renal replacement therapy (RRT) in managing ARDS and AKI secondary to scrub typhus. However, these are not substitutes for timely etiology-specific treatment. In Case 2, despite maximal supportive care-including mechanical ventilation, vasopressors, and empirical antimicrobials-delayed initiation of doxycycline precluded recovery. This mirrors broader epidemiologic observations that scrub typhus, once considered a rural disease, increasingly affects urban dwellers, travelers, and peri-urban populations, often leading to delays in diagnosis due to lack of clinical suspicion (Singh and Panda, 2024[14]). Moreover, the lethality of scrub typhus in young, otherwise healthy individuals highlights the need for better awareness among primary and emergency care providers. Mortality rates in complicated scrub typhus range from 10-30 %, with the highest risk in patients with delayed diagnosis, CNS or pulmonary involvement, or multiorgan failure (Jamil et al., 2014[7]; Prakash, 2017[11]). Despite the growing burden, scrub typhus remains under-recognized in national fever algorithms and is seldom included in syndromic surveillance programs outside northeast India.

Finally, our case underlines the need for scalable point-of-care diagnostics and broader antimicrobial coverage in fever algorithms in endemic settings. Development of multiplex platforms capable of detecting *O. tsutsugamushi* alongside dengue, malaria, and leptospira would dramatically improve early detection. Public health interventions must also prioritize vector control and community education in high-risk regions during post-monsoon months. Our series is limited by its small sample size, single-center design, absence of PCR confirmation, and lack of autopsy in the fatal case. Potential co-endemic infections cannot be fully excluded.

In conclusion, scrub typhus continues to present formidable diagnostic and therapeutic challenges in India. Clinical vigilance for eschar, early empirical therapy with doxycycline and/or azithromycin in classical febrile syndromes, and prompt escalation to combination therapy in severe disease remain key pillars of management. The fatality in Case 2 starkly reminds us that age and immunocompetence offer no protection against this elusive but treatable infection-if not diagnosed in time.

## Conclusion

Scrub typhus continues to challenge clinicians due to its mimicry, cross-reactivity, and variable organ involvement. The presence of eschar, CNS signs, refractory ARDS, or unexplained AKI in a febrile patient must raise suspicion in endemic zones. Early doxycycline, rigorous physical examination, and multi-organ vigilance remain the cornerstones of management. As these cases demonstrate, the line between full recovery and fatal outcome may hinge on a single clue: the overlooked eschar.

## Declaration

### Author contributions

YS, AS: Conceptualization, supervision, validation, AK, PC, NU: writing-original draft, visualization, project administration, writing - review & editing. PNC, SC, HC: Writing-original draft, review & editing.

### Funding

The authors did not receive support from any organization for the submitted work. 

### Financial interests

The authors declare they have no financial interests. The authors have no relevant financial or non-financial interests to disclose.

### Institutional Review Board statement

Ethical review and approval were not required for this case series as determined by the University Ethics Committee of Subharti Medical College, Swami Vivekanand Subharti University, Meerut and Department of Medicine, Subharti Medical College, Meerut (exemption letter dated 15 Jul 2025).

### Informed consent statement

Written informed consent for publication (including clinical images) was obtained from patients; for the deceased patient, consent was obtained from the next of kin.

### Data availability statement

Data is available on request from the corresponding author.

### Conflict of interest

The authors declare that no funds, grants, or other support were received during the preparation of this manuscript.

### Declaration on the use of Artificial Intelligence

No AI tools were utilized for content generation, data analysis, or interpretation. AI was strictly limited to grammatical refinement and data visualization. All final content was reviewed and verified by the authors, who maintain full responsibility for the integrity and accuracy of the work.

## Figures and Tables

**Table 1:**
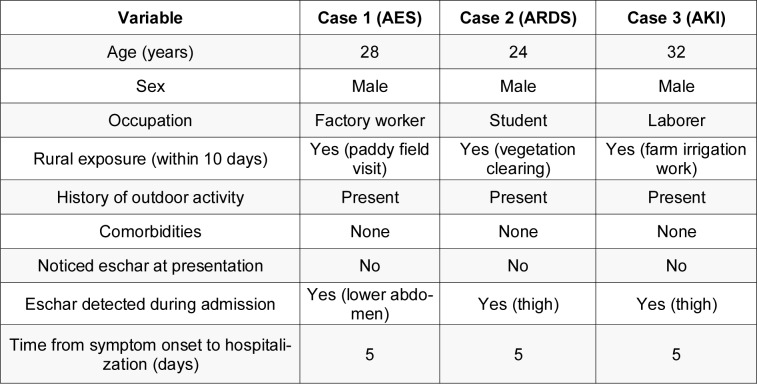
Baseline demographics and exposure history of the patients

**Table 2:**
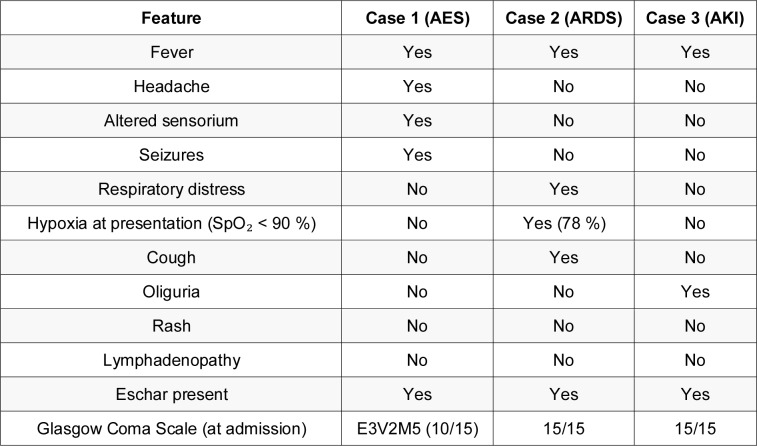
Presenting clinical features and organ involvement

**Table 3:**
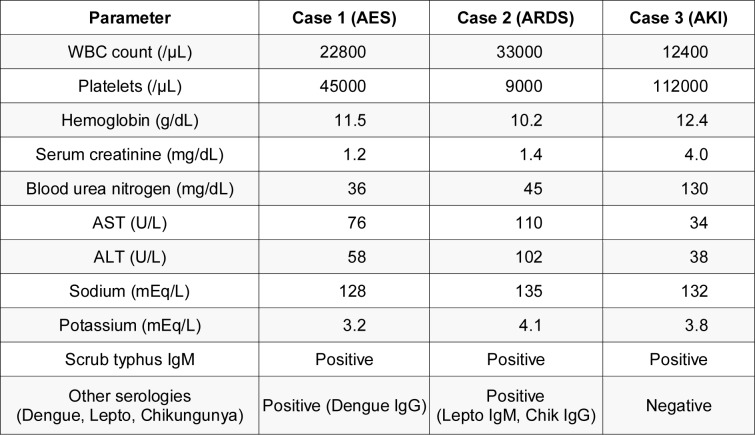
Laboratory parameters at admission

**Table 4:**
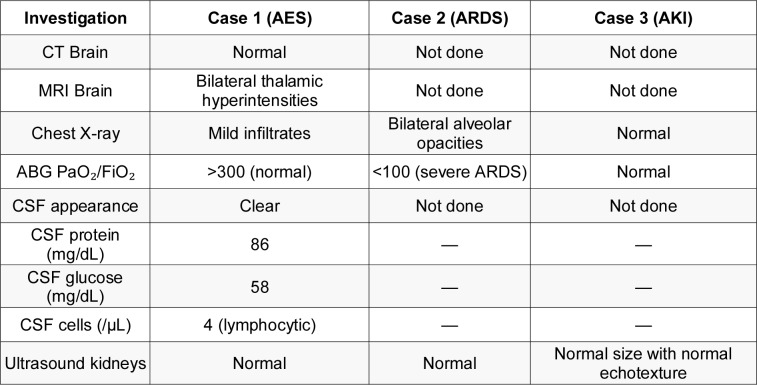
Imaging and CSF findings

**Table 5:**
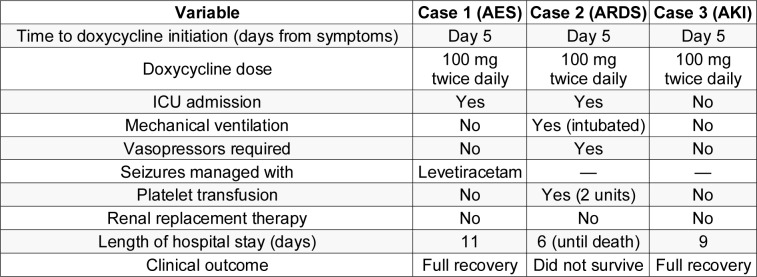
Treatment, clinical course, and outcomes

**Table 6:**
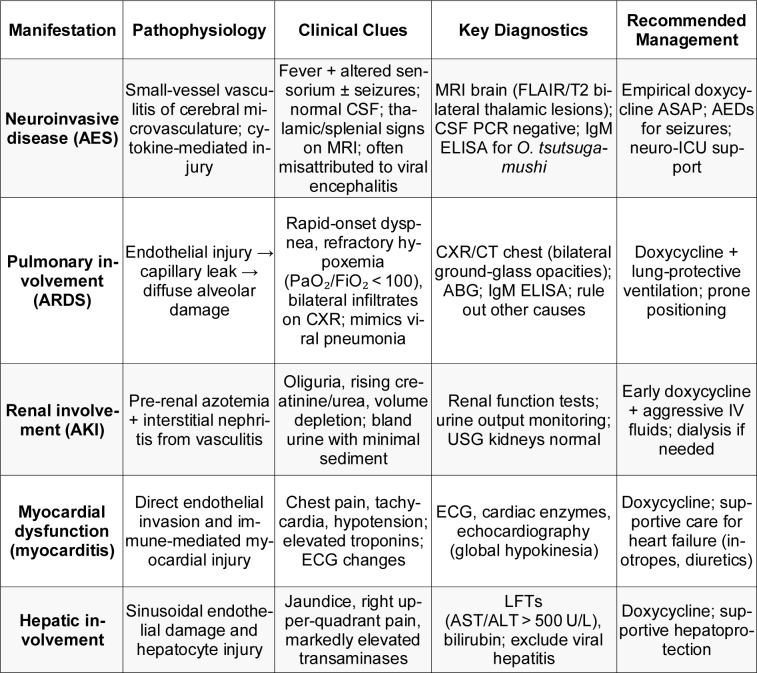
Atypical organ-specific manifestations of scrub typhus
